# Endocrine disruptors and pregnancy

**DOI:** 10.55730/1300-0144.6123

**Published:** 2025-11-26

**Authors:** Betül YİĞİT YALÇIN, Gamze BİLİK OYMAN, Ayşe KUBAT ÜZÜM

**Affiliations:** Department of Endocrinology, İstanbul Faculty of Medicine, İstanbul University, İstanbul, Turkiye

**Keywords:** Endocrine disruptors, pregnancy, fetus, lactation

## Abstract

**Background/aim:**

Sensitivity to endocrine disruptors is higher in early life. Endocrine disruptor chemicals can be passed from pregnant women to their babies through the placenta or breast milk during lactation, leading to long-term and potentially permanent adverse effects.

**Materials and methods:**

This review evaluates the effects of endocrine-disrupting chemicals (EDCs) on pregnancy by summarizing findings from experimental and observational studies. Exposure routes, reproductive outcomes, fetal development implications, and potential preventive strategies are analyzed.

**Results:**

Exposure to EDCs during pregnancy has been linked to various complications, including infertility, implantation defects, premature birth, spontaneous abortions, gestational hypertension, and gestational diabetes. Intrauterine exposure to these chemicals may lead to metabolic disorders, congenital anomalies, low birth weight, and delayed physical and mental development in offspring depending on the level and timing of exposure.

**Conclusion:**

Due to the significant impact of endocrine disruptors on maternal and fetal health, it is critical to implement protective measures to reduce exposure during pregnancy and lactation. Increased awareness and preventive strategies can help mitigate adverse effects.

## 1. Introduction

Endocrine disruptors are substances that have adverse effects on the endocrine system by altering the synthesis, release, transport, metabolism, binding, and excretion of hormones. The effects of endocrine-disrupting chemicals (EDCs) can be seen in healthy organisms but can also have negative consequences in subsequent generations. The age at which an individual is exposed to EDCs is important, and the effects of exposure in the intrauterine period, childhood, or adulthood vary. The developing organism is more sensitive to EDCs, and exposure to EDCs early in life can cause irreversible effects. Some endocrine disruptors can accumulate in body fat in pregnant women, cross the placenta, and affect the fetus. Some endocrine disruptors may pass from breast milk to the infant and cause adverse effects [1]. However, observational and experimental studies show that exposure to EDCs negatively affects fertility by interfering with processes such as folliculogenesis, steroidogenesis, ovulation, implantation, and pregnancy [2]. These substances can cause problems in reproductive health, such as precocious puberty, infertility, early ovarian failure, disturbances in postnatal ovarian structure/function, endometriosis, fibroids, and adverse pregnancy outcomes [1]. EDCs can disrupt intrauterine implantation early in pregnancy, causing implantation defects [3]. EDC exposure during pregnancy can cause irreversible modification of molecular, cellular, and hormonal signaling pathways, predisposing the fetus to organ dysfunctions and chronic diseases in adulthood [4].

There are many routes of exposure for EDCs to pass to the baby. They can be transmitted through the skin, by inhalation, orally through food, transplacentally from the mother to the baby, or through breast milk. Sometimes chemicals can pass directly from the mother to the baby [1].

Results of some animal and observational studies investigating the adverse effects of frequently encountered EDCs on the mother and the fetus are summarized in Table.

## 2. Adverse effects of frequently encountered EDCs on mother and fetus

### 2.1. Bisphenol

Bisphenol A (BPA) has a wide range of uses, including epoxy resins, plastic bottles, food packaging, toys, and the linings of canned foods. It can pass into food and water with high temperatures, and because of repetitive use, exposure to these products is extremely high. BPA has also been detected in breast milk [1]. Experimental studies show that BPA decreases fertility. In an animal study, administration of BPA to fertilized female mice on days 0 and 1 of pregnancy decreased the number of implantation sites [5]. In another study, pregnant rats exposed to BPA during the neonatal period had fewer implantation sites [1,6–7]. Prenatal BPA exposure also affected subsequent generations. Decreased pregnancy indices in the first filial (F1) and second filial (F2) generations, and decreased ability to maintain pregnancy in the third filial (F3) generation, were observed in mice with prenatal BPA exposure [8]. In a study of women treated with in vitro fertilization, an increase in implantation failure was observed as urinary BPA levels increased [9]. In a cohort study of women undergoing pregnancy follow-up, it was found that the risk of miscarriage increased as maternal BPA levels increased [10]. Another study showed that BPA levels were higher in infertile women than in fertile women [11]. Studies on whether BPA reduces the duration of pregnancy are not clear. In animal experimental studies, exposure to BPA did not induce preterm birth. However, in a case-control study conducted in pregnant women, it was shown that high urinary BPA levels increased the risk of preterm birth [12]. In a Dutch population-based prospective cohort study, the authors demonstrated an association between prenatal BPA exposure and impaired intrauterine growth. BPA exposure caused a decrease in maternal and fetal weight, and high concentrations of maternal urinary BPA caused a decrease in fetal head circumference, which may adversely affect fetal growth [13]. On the contrary, in a multicenter study conducted in Korea, it was reported that fetal BPA exposure increased birth weight and height in male newborns, and that anthropometric measurements varied by sex [14]. Intrauterine BPA exposure also affects the urogenital system. In animal studies, prenatal BPA exposure has been shown to reduce anogenital distance in male mouse offspring and to alter ovarian function in female mice [15]. In an animal study examining the relationship between prenatal exposure to BPA and obesity, sex-specific changes were observed in mice exposed to nontoxic doses of BPA during pregnancy and lactation. While an increase in body and liver weights was observed in male mice, no change was observed in adipose tissue, whereas a dose-dependent decrease in body weight, liver, muscle, and adipose tissue weights was found in female mice [16]. In a study conducted with toxic levels of BPA exposure, weight loss was observed [1]. Many studies have investigated prenatal and perinatal exposure to BPA and glucose balance. Prenatal exposure to BPA (10 μg/kg/day) caused insulin resistance, hyperinsulinemia, and glucose intolerance. Different doses of BPA (five to 50,000 μg/kg/day) administered to pregnant mice resulted in insulin resistance and decreased serum adiponectin (ApN) levels at all doses. Hyperinsulinemia was observed at the lowest dose level, while weight gain was observed only at 500 μg/kg/day exposure [17]. Another study showed that perinatal exposure (3.5 μg/kg/day) caused glucose intolerance in 3-month-old male offspring but not in female offspring [18]. The mechanisms by which perinatal exposure causes insulin resistance have been explained. Perinatal exposure to BPA is thought to cause deoxyribonucleic acid (DNA) methylation, leading to decreased glucokinase expression and increased peroxisome proliferator-activated receptor gamma expression. Dietary BPA exposure during pregnancy and lactation may affect cortical development, mimicking thyroid resistance syndrome. It may increase serum total thyroxine (TT4) levels without altering thyroid-stimulating hormone. BPA acts selectively as an antagonist on thyroid hormone receptor beta, disrupting the negative feedback mechanism. In a study conducted in mice, BPA exposure during pregnancy and lactation increased TT4 levels on day 15, which returned to normal on day 35 [19]. Accumulation of BPA in the placenta causes DNA methylation defects and alterations in gene expression, and these epigenetic changes affect placental function and fetal health [1].

### 2.2. Phthalates

Phthalates are widely used in personal care products, perfumes, creams, lotions, shampoos, polyvinyl chloride plastics, sports drinks, infusion bags, ventilation materials, hemodiafiltration, nasal cannulas, masks, nasogastric catheters, and urethral catheters [1]. Due to their structure, they can spread easily into the environment. Clinical studies show that phthalates can be detected in serum, urine, and breast milk. Exposure to di(2-ethylhexyl)phthalate (DEHP), one of the major phthalates, ranges from three to 30 μg/kg/day [1]. In a study examining pregnancy and phthalate levels, a relationship was found between urinary phthalate levels and pregnancy losses [20]. In another study, fertile couples and couples admitted to the clinic due to infertility were compared, and it was observed that phthalate levels in urine samples of infertile couples were higher than those of fertile couples [11]. Animal studies also confirm that phthalate exposure reduces fertility. In pregnant mice, exposure to phthalate disrupted implantation in the endometrium and caused pregnancy loss [21]. However, animal experiments with polypropylene and polyethylene showed no effect on the uterus [22]. Prenatal exposure to DEHP increased the length of pregnancy in mice, and in another study, pregnancy loss occurred in all mice [23,24]. Whether phthalate exposure causes preterm birth has not yet been clearly established. It is thought that phthalate exposure during pregnancy impairs the function of the placenta by affecting trophoblast differentiation and placental steroidogenesis, and this may increase the risk of preterm birth. A study conducted in Mexico found a positive correlation between some phthalate metabolites and the risk of preterm birth [25]. In another study, metabolites of mono(2-ethylhexyl) phthalate (MEHP), mono-(2-ethyl-5-oxohexyl) phthalate, mono(2-ethyl-5-carboxypentyl) phthalate, mono-n-butyl phthalate (MBP), and mono-(3-carboxypropyl) phthalate were shown to increase the risk of preterm birth [26]. Phthalate exposure during pregnancy has been associated with decreased placental weight and impaired fetal growth [27]. In a study conducted in Japan, the effects of phthalate exposure in pregnant women on genital system development in male newborns were examined. It was shown that high levels of maternal urinary MEHP caused a decrease in the anogenital distance in male newborns and negatively affected reproductive development in males [28]. In addition, a positive correlation was observed between MBP levels in amniotic fluid and decreased anogenital distance in female newborns [29]. An increased risk of preterm birth was reported with phthalate exposure in a prospective, observational cohort study [30]. In animal studies, it was also found that male and female mice exposed to DEHP in the intrauterine and lactation periods had changes in estrogen synthesis and decreased reproductive performance [31]. At the same time, exposure to di-n-butyl phthalate (DBP) in the intrauterine period caused fetal weight gain in female rats [32]. In a prospective study conducted in Korea, exposure to phthalates in the prenatal period caused low performance in mental development and psychomotor index at 6 months in male infants [33]. Masculine play behavior decreased in boys exposed to phthalates in the prenatal period [34]. In an animal study evaluating mammary development and phthalate exposure during pregnancy, it was observed that mammary development was poor in adult female offspring of rats exposed to DBP from late pregnancy through lactation. Butyl benzyl phthalate exposure during pregnancy has been shown to cause a marginal acceleration in the mammary growth but to delay the onset of puberty [1,35].

### 2.3. Pesticides

Pesticides are commonly detected in contaminated water and soil associated with grass and crops. Exposure can occur through the digestive, respiratory, and dermal routes.

Several recent animal studies have demonstrated that pesticide exposure adversely affects fertility and infertility [1]. In animal studies with pesticides, methoxychlor has been shown to decrease fertility by increasing uterine mass in nonpregnant rats and decreasing the number of implantation sites and the number of newborns in pregnant rats [36]. Synthetic insecticides have been reported to impair implantation in pregnant rats [37]. Organophosphate pesticides have also been found to cause DNA and ribonucleic acid (RNA) damage in testicular tissue, impaired sperm morphology, movement, and function, decreased in vitro fertilization potential, and embryogenic degeneration in rats [38]. In women receiving fertility treatment, hexachlorobenzene levels were found to be significantly higher, and this was explained by failed implantation [39]. In a study conducted in Africa, it was found that the rate of spontaneous abortion was higher in women who were engaged in agriculture and used pesticides in the first trimester of pregnancy [40]. In another study conducted on pregnant women, high concentrations of 2,2′,4,4,4′,5,5′-hexachlorobiphenyl and p,p’-dichlorodiphenyldichloroethylene were associated with increased fetal loss [41]. Studies on pregnant women have shown that pesticide exposure has adverse effects on the duration of pregnancy. Atrazine (2-chloro-4-ethylamino-6-isopropylamino-s-triazine) (ATR) levels in drinking water in the state of Kentucky between 2000 and 2008 were examined, and it was shown that the risk of preterm birth increased in women in regions with the highest ATR levels [42]. Furthermore, a study in France found that chlordecone exposure in women was related to the risk of preterm birth. [43]. However, in some studies, pesticide exposure was found not to affect the duration of pregnancy. Studies with hexachlorobenzene in Spain and ATR in India have shown that neither shortens labor duration. Since endocrine-disrupting pesticides affect gene expression, in utero and early childhood exposures have more harmful effects than adult exposures [44]. Second-trimester methyl bromide exposure has been shown to cause low birth weight, birth length, and head circumference in the newborn [1]. In a study of 2246 female farmers, the association between maternal pesticide use and birth weight was examined, including 27 pesticides. No association was found between pesticide exposure in the first trimester and birth weight, but regular pesticide exposure was shown to cause low birth weight. Studies show that ATR exposure is associated with low head circumference but not with congenital anomalies. Children exposed to BPA and pesticides in the first trimester of pregnancy were examined between two and eight years of age. It was found that body mass index and waist circumference increased [4]. Environmental pesticide exposure was found to affect the placenta and increase IL-13 expression, an antiinflammatory cytokine [45]. Animal studies also confirm that pesticide exposure is associated with decreased birth weight and length [1]. In a study conducted after an explosion at a pesticide factory in Italy, it was observed that men who were breastfed after the explosion developed a permanent decrease in sperm quality in the later period [36]. Some types of pesticides have been associated with decreased cognitive function. One study found that children with high levels of chlorpyrifos in umbilical cord blood had lower intelligence quotient (IQ) scores and poorer working memory at age 7 [46]. In pregnant women living in a region where organophosphate pesticides were used, it was shown that the full-scale IQ levels of the children of mothers with high urinary dialkyl phosphate metabolite levels were seven points lower. In another study, an increase of 60% in autism spectrum disorder and 150% in developmental delay disorder was observed in pregnant women living in an area where pesticides were used [47]. An animal study examined the effect of ATR exposure during pregnancy on mammary development in offspring and showed that ATR exposure weakened mammary gland development in female offspring [48]. A human study demonstrated that dichlorodiphenyltrichloroethane exposure during pregnancy increased the risk of adult breast cancer in female newborns, independent of maternal breast cancer history [49]. Chronic exposure to chlordecone, an organochlorine pesticide, has been reported to cause gestational hypertension and gestational diabetes [50].

### 2.4. Perfluorinated compounds

Perfluorinated compounds have widespread uses, such as in fire-extinguishing products, electrical cables, and dental treatment materials.

Studies have shown that exposure to perfluorooctanoic acid (PFOA) and perfluorooctane sulfonate (PFOS) adversely affects pregnancy. Serum PFOA and PFOS have been shown to cause gestational hypertension. In addition, a metaanalysis in rodents found a decrease in birth weight [1]. In the offspring of female mice exposed to PFOA for 17 days of gestation, it was observed that body mass, serum insulin, and leptin values increased in the 20- to 40-week adult period [51]. PFOA is known to cross the placenta. It has been found that girls with higher PFOA exposure in the prenatal period have a later time to puberty than those with lower exposure. However, animal studies have demonstrated that PFOA exposure does not affect the timing of puberty but alters breast development [1]. Studies suggest that exposure to PFOA is possible through breast milk. One study found a significant correlation between serum PFOA levels measured in childhood girls and duration of breastfeeding [52]. Both PFOS and PFOA are thought to disrupt lactation and cause shorter breastfeeding duration [53]. Animal studies have revealed that PFOA alters the timing and function of mammary development. Exposure of pregnant mice to PFOA also caused lactation failure and increased offspring mortality [1]. White et al. examined the differentiation of PFOA exposure on the mammary gland in F1 and F2 female mice and found that exposure slowed mammary gland development [54]. In another animal experiment in which PFOA exposure was induced at lower doses than in this study, PFOA was administered to mice at different gestation periods, and, similarly, it was observed that mouse mammary gland development was inhibited [55].

### 2.5. Polybrominated diphenyl ethers

Polybrominated diphenyl ethers (PBDEs) are flame retardants used in a range of applications, including clothing, plastics, the automotive industry, and insulation materials. PBDEs have been found in human serum, cord blood, and breast milk [1].

In a study, the relationship between PBDE level in cord blood and full-scale IQ level was examined. It was observed that children with higher levels of PBDEs in cord blood scored lower on tests measuring mental and physical improvement [56]. There are also different studies reporting that PBDE is negatively correlated with cognitive functions [1].

### 2.6. Dioxin

Dioxin is found in chlorine bleaching of paper pulp in industrial processes, in the production of some herbicides and pesticides, and in animal feed.

Dioxin causes disorders in reproductive function. 2,3,7,8-tetrachlorodibenzo-p-dioxin (TCDD) levels have been positively correlated with increased infertility rates. In studies on mice, TCDD exposure has been reported to shorten the gestation period and, in the next three generations, increase inflammation [1]. In another study, TCDD exposure in mice decreased offspring survival [57]. In another study, in utero TCDD exposure was shown to induce transgenerational effects, leading to premature puberty in the F3 generation of female mice [58]. In a prospective study, low birth weight was observed in male infants prenatally exposed to polychlorinated dibenzodioxin and polychlorinated dibenzofurans [59]. In a study conducted in Japan, concentrations of polychlorinated dibenzodioxins and dibenzofurans in breast milk showed inverse relationships with newborn length and head circumference. This suggests that maternal dioxin exposure adversely affects fetal growth [60].

### 2.7. Parabens

Parabens are widely found in products such as personal care supplies (toothpaste, tooth-polishing powder, sunscreen oils, skin cleansers, roll-ons, and soaps), cosmetics (cream, foundation, powder, eye shadow, mascara, makeup remover, lipsticks) and medicines in the form of ointments, eye, ear and nose drops, ovules, food additives [61].

Experimental studies suggest that parabens may contribute to ovarian aging by reducing the ovarian reserve, which may be associated with early menopause and infertility in women [62]. Paraben exposure in the prenatal period may disrupt thyroid hormone balance and cause high birth weight in boys [63]. One study showed that cord serum leptin levels were altered in infants exposed to parabens in utero, suggesting that parabens may affect fetal growth [64]. Another study exploring the impact of paraben exposure on pregnancy discovered a positive correlation between exposure and gestational diabetes [65].

### 2.8. Arsenic

Arsenic is a naturally occurring element in soil. Exposure can result from agriculture and mining. It is also used in wood preservation products, electronic equipment, glass, and ceramic products [66].

In a study on pregnant rats, arsenic exposure was shown to increase gonadotropin-releasing hormone levels and cause early puberty in their offspring [67]. In a human study, arsenic exposure was associated with placental oxidative damage, which may contribute to adverse pregnancy outcomes [68].

### 2.9. Polycyclic aromatic hydrocarbons

Polycyclic aromatic hydrocarbons (PAHs) can occur naturally, for instance, from volcanic eruptions and forest fires. They can be released from industrial areas such as asphalt production, oil refineries, cement factories, and aluminum, iron, and steel factories [69]. They can also be found in heat-treated foods such as roasted coffee, tea, and vegetable oil, as well as in their packaging.

Due to their lipophilic structure, PAHs can cross the placenta and cause teratogenic effects. They can cause conditions such as neural tube defects, gastroschisis, and cleft palate. Exposure to high doses of PAHs during pregnancy can cause low birth weight, prematurity, low IQ, behavioral problems, and childhood asthma [70–75]. As urinary concentrations of 1-hydroxypyrene, a metabolite of polycyclic aromatic hydrocarbons, increase, birth weight, birth length, and head circumference decrease [30].

### 2.10. Diethylstilbestrol

Diethylstilbestrol (DES) is a nonsteroidal estrogen used between 1938 and 1971 to improve pregnancy outcomes [76]. However, an increase in the rate of vaginal clear cell adenocarcinoma was found in exposed girls, and it was discontinued [77]. Exposure to DES in the intrauterine period has been shown to increase the risk for disorders of the genitourinary system, some types of cancer in children of both sexes, and cryptorchidism (in boys) [1]. Another study involving DES indicated a link between prenatal exposure to DES and the likelihood of experiencing early menopause [78]. In animal studies, DES has been found to affect the mammary glands, and high doses of prenatal and lactational DES exposure increased mammary growth in offspring and reduced the number of terminal end buds [1].

### 2.11. Mixtures of EDCs

People are exposed to many chemical combinations throughout their lives. There is a need for studies on these combinations as much as single-compound studies on endocrine disruptors [1]. In an animal study, a mixture of DEHP, BPA, polychlorinated biphenyl-153, and TCDD was added to the diets of mice from before pregnancy through pregnancy and lactation. Some sex-dependent metabolic changes were observed in mouse offspring. In males, this exposure decreased hepatic total cholesterol levels but did not affect glucose tolerance. However, impaired glucose tolerance was observed in female mice. This study differed from others in examining not a single exposure to EDCs but a chemical mixture [79].

Figure provides a schematic representation of the possible impacts of EDCs on maternal and fetal systems during pregnancy.

## 3. Conclusion

More comprehensive animal and human studies are still needed to reveal the health problems that exposure to endocrine disruptors in the intrauterine period may cause in adulthood. It is much more difficult to evaluate the detrimental effects of EDCs on the mother and the fetus during pregnancy, especially considering factors such as the difficulty of obtaining blood and urine samples during pregnancy and the unclear timing of exposure.

It is very important to take protective measures for pregnant women and children and to raise awareness. Since sensitivity to endocrine disruptors is higher in the intrauterine period and early life, women during pregnancy and lactation should take some precautions. During these periods, cotton clothing should be used instead of synthetic and dyed clothes. The use of cosmetic products should be reduced. Plastic containers should be avoided whenever possible, and if used, they should not be exposed to heat. When buying food products, attention should be paid to ingredients and products that do not contain EDCs, and these should be preferred whenever feasible.

## Figures and Tables

**Figure:**
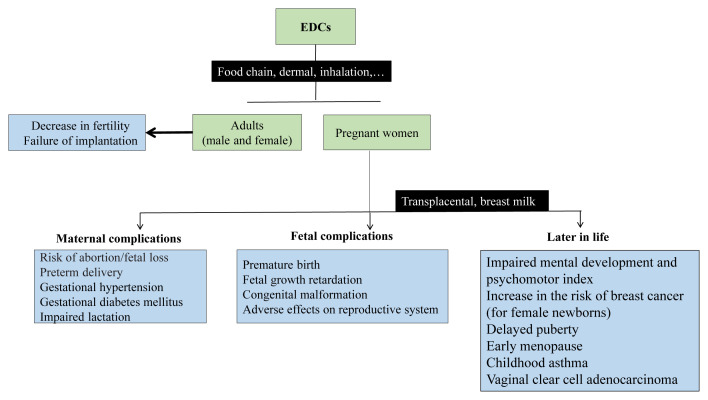
Overview of the potential effects of EDCs during pregnancy. The figure illustrates exposure routes, maternal and fetal complications, and the possible long-term health outcomes.

**Table:** Effects of EDCs on mother and fetus.

EDCs	Effect on	Animal models	Observational

**BPA**	**Mother**	Failure of implantation, infertility	Failure of implantation, infertility, risk of abortion
	**Fetus**	Insulin resistance and decrease in ApN, increase in TT4, sexually dimorphic changes; alteration in AGD (male) and ovarian function (female)	Premature birth, FGR

**Phthalates**	**Mother**	Failure of implantation, infertility, fetal loss, decreased reproductive performance	Infertility, fetal loss
	**Fetus**	Premature birth, fetal weight gain, poor mammary gland development, delayed puberty	Premature birth, FGR, adverse effects on the reproductive system, impaired mental development and psychomotor index

**Pesticides**	**Mother**	Failure of implantation, infertility, low fertilization potential	Failure of implantation, infertility, spontaneous abortions, fetal loss, GHT, GDM
	**Fetus**	Fetal growth retardation, poor mammary gland development	Risk of preterm birth, low birth weight, FGR, decreased sperm quality, decreased IQ, autism spectrum disorder, and increased risk of breast cancer (female)

**PFCs**	**Mother**	Impaired lactation	GHT, impaired lactation
	**Fetus**	Decreased birth weight, increased body weight, serum insulin and leptin levels, and poor mammary gland development	Delayed puberty

**PBDE**	**Fetus**	N/A	Regression of mental and physical development

**Dioxin**	**Mother**	Shortened gestation, fetal loss	Infertility
	**Fetus**	Potential transgenerational effects: premature puberty in F3 generation (females)	FG, low birth weight (male)

**Parabens**	**Mother**	Potential infertility	GDM
	**Fetus**		Increased cord serum leptin levels, disruption of thyroid hormone balance, and increased birth weight

**Arsenic**	**Mother**		Placental oxidative damage
	**Fetus**	Early puberty	

**PAHs**	**Fetus**	N/A	Neural tube defects, gastroschisis and cleft palate, low birth weight, prematurity, low IQ, behavioral problems, childhood asthma

**DES**	**Fetus**	Increased mammary growth and decreased number of TEB	Vaginal clear cell adenocarcinoma, early menopause, cryptorchidism (males)

Abbreviations: FGR, fetal growth retardation; ApN, adiponectin; TT4, total T4; AGD, anogenital distance; PBDE, polybrominated diphenyl ethers; GDM, gestational diabetes mellitus; GHT, gestational hypertension; DES, diethylstilbestrol; PAHs, polycyclic aromatic hydrocarbons; PFCs, perfluorinated compounds; BPA, bisphenol A; TEB, terminal end buds; N/A, not available
